# Effectiveness of a Nurse-Led Ambulatory Care Clinic in Reducing Emergency Department Visits and Hospitalizations

**DOI:** 10.5811/westjem.53190

**Published:** 2026-09-05

**Authors:** Seung-Yup Lee, Reid M. Eagleson, Larry R. Hearld, Madeline J. Gibson, Allyson G. Hall, Michael J. Mugavero, Greer Burkholder, Kimberly L. Payne, William M. Brown, Lauren M. Epp, Laurie Hunter, Corey T. Spraberry, Kristine R. Hearld

**Affiliations:** * University of Alabama at Birmingham, Department of Health Services Administration, School of Health Professions, Birmingham, Alabama; † University of Alabama at Birmingham, Center for Outcomes and Effectiveness Research and Education, Birmingham, Alabama; ‡ University of Alabama at Birmingham, Department of Medicine, Birmingham, Alabama; § University of Alabama at Birmingham Medicine, Birmingham, Alabama

## Abstract

**Introduction:**

Gaps in care integration and coordination contribute to emergency department (ED) crowding and preventable hospitalizations, necessitating innovative solutions to improve care transitions and reduce acute care use. This study evaluates whether attendance at a nurse-led ambulatory intermediate care clinic (AICC) reduces three-month ED visits and hospitalizations compared to missed AICC appointments.

**Methods:**

This retrospective cohort study at a single academic center analyzed AICC appointments from the first three years of clinic operations using multivariable logistic regression models, controlling for demographic, socioeconomic, and clinical factors. Our primary outcome measure was an ED visit within three months of the AICC appointment; our secondary outcome measure was hospitalization within three months. Robustness was assessed using Poisson, negative binomial, and zero-inflated models.

**Results:**

Among 2,698 appointments analyzed (mean age 51.5 years; 49.7% female), patients who attended their AICC appointment had a lower absolute ED visit rate compared with those who missed it (250/2,389 [10.5%] vs 47/309 [15.2%]; risk difference, 4.75%; 95% CI, 0.56–8.93%; *P* = .03). Missing an AICC appointment was significantly associated with greater odds of an ED visit (OR, 1.54; 95% CI, 1.07–2.21; *P* = .02). Hospitalization rates were 47.3% versus 53.4% (*P* = .04), although missing an AICC appointment was not significantly associated with increased hospitalizations (OR, 1.28; 95% CI, 1.00–1.64; *P* = .05). Findings were consistent across robustness analyses.

**Conclusion:**

The ambulatory intermediate care clinic model was associated with fewer ED visits but not reduced hospitalizations, suggesting that intermediate care can improve timely access and address acute care needs but may be insufficient to influence more complex drivers of inpatient admissions. Future research should explore strategies to enhance coordination and optimize the role of intermediate care within broader health system workflows.

## INTRODUCTION

Emergency departments (ED) and hospitals are increasingly confronting the challenge of crowding.^1^–^3^ This issue extends beyond the boundaries of individual facilities, emerging as a systemic problem that is influenced by and affects the coordination and capacity of the broader landscape of healthcare delivery.^4^ In this context, ambulatory care settings often encounter patients whose health concerns exceed the capacity of these clinics, necessitating referrals to EDs or hospitals.^5^,^6^ As a result, primary care clinics have become a notable source of referrals, unintentionally contributing to the crowding in both EDs and hospitals.^7^ This trend underscores the complex interplay between various levels of healthcare provision (eg, nonacute versus acute) and the need for integrated solutions to address systemic crowding.

In response to this challenge, the concept of intermediate care has been proposed as a viable solution.^8^–^11^ Intermediate care, first introduced in healthcare systems in the United Kingdom,^8^,^12^ aims to provide a level of care that sits between the intensity of the ED or hospital and the basic care offered in outpatient settings.^13^ A large academic health system has pioneered a nurse-led ambulatory intermediate care clinic (AICC) with the objective of mitigating ED congestion and reducing avoidable hospital admissions. The nurse-led AICC is designed to cater to patients requiring more than primary care but less than acute hospital or ED care, focusing on balancing efficiency and quality of care. The AICC operates same-day referrals from outpatient clinics and has demonstrated measurable success during its first three years of operation, including a consistent increase in the volume of referrals and the ability to manage presenting patients without requiring same-day transfer to the ED.^14^

The AICC manages patients requiring services such as intravenous fluid administration, diagnostic workup for acute symptom exacerbation, medication adjustments, and monitoring for conditions such as chest pain, shortness of breath, or uncontrolled hypertension that exceed primary care capacity but do not yet warrant ED-level intervention. Conceptually, by addressing access barriers—such as affordability (lower copays than ED visits), availability and accommodation (avoiding long waits in overcrowded EDs), and accessibility (co-location with referring clinics)^15^—the AICC aims to address unmet health needs before they escalate to conditions requiring ED visits or hospitalizations.

However, the effectiveness of such intermediate care remains an area of exploration. Traditional evaluations of intermediate care often focus narrowly on within-clinic metrics, such as number of referrals, appointment attendance rates, and outcomes specific to those who received care.^9^,^16^ While these approaches provide valuable initial insights into the clinic’s role in care delivery, they fail to capture the broader value of intermediate care clinics within the larger healthcare network.^17^ This limitation restricts the understanding of their systemic roles and contributions. Moreover, such evaluations performed at the clinic level, rather than the patient level, often overlook diverse patient characteristics—such as demographic factors, socioeconomic status, and clinical conditions—which critically influence a patient’s access to care and use patterns.

**Population Health Research Capsule**

What do we already know about this issue?

*Intermediate ambulatory care clinics may reduce emergency department (ED) crowding, but nurse-led models and their impact on ED visits is limited*.

What was the research question?

*Does attending a nurse-led intermediate care clinic reduce three-month ED visits and hospitalizations?*

What was the major finding of the study?

*Missing an ambulatory intermediate care clinic appointment: 54% greater odds of ED visit (OR, 1.54; 95% CI, 1.07–2.21; P = .02)*

How does this improve population health?

*Nurse-led intermediate ambulatory care clinics can reduce ED use by providing timely access to care that bridges primary care and emergency services*.

This study fills these gaps through an in-depth, patient-level evaluation of the AICC, focusing on reducing ED visits and hospitalizations. We hypothesized that patients who received care in the AICC would have a lower likelihood of experiencing both an ED visit and an inpatient hospital stay compared with those who did not receive care in the clinic.

## METHODS

### Study Setting

This study took place in the nurse-led AICC within a large, multiclinic outpatient center in a key urban area in the United States. The outpatient center offers a wide range of outpatient services, from primary care to specialties like cardiovascular, neurology, oncology, and diabetes management. Additionally, a nearby complementary clinic extends the capacity for outpatient care. Combined, these two clinics treat over 2,000 patients per day. The AICC, implemented in May 2020, functions as a same-day referral clinic where clinicians from both outpatient facilities can call to discuss concerns regarding their patients and receive feedback on patient acceptance.^14^ The AICC operates Monday–Friday, 8 am to 8 pm and provides same-day referrals for patients requiring a higher level of acuity, treating patients and discharging them home with follow-up care in the outpatient setting or transferring them to the ED as clinically indicated. Led by two registered nurse practitioners, the AICC is staffed by four registered nurses, a pharmacist, and a patient encounter specialist. It also has access to laboratory, imaging, and other services to assess and treat the patients. In collaboration with the referring physician, the AICC determines whether the patient can be safely discharged home or requires transfer to the ED.

### Data

Data were extracted from the electronic health record system by the institutional research and informatics service center using a structured approach consistent with recommended practices for retrospective medical record-based research.^18^ Data collected included appointment characteristics and patient demographics and clinical information. Appointment characteristics, deidentified to protect patient privacy, encompassed both the scheduling and actual appointment dates and times. Our dataset also covers pertinent comorbidities and the appointment status (arrived, no show, canceled, or rescheduled). We combined the “cancelled” and “no show” categories into one category, ie, “missed,” for a clear binary representation, forming a binary category (“arrived” versus “missed”), especially considering the relatively low frequency of no-shows (0.6%).

Demographic and socioeconomic details provided are the patient’s city, state, Zip code, age, sex, ethnicity, race, marital status, preferred language, and primary insurance provider. These pieces of information are vital for analyzing patients’ healthcare access and use, as well as for identifying areas where there may be disparities in care delivery.^19^,^20^ The independent variable was appointment status; demographic, socioeconomic, and clinical variables were incorporated as covariates. To test the hypotheses, we used two dependent variables: (1) ED visits within three months following each patient’s AICC appointment and (2) hospitalizations within the same timeframe. Because missing values were expected to be minimal, the degree of missingness was evaluated and summarized in the results narrative instead of being examined in detail.

This study was approved by the Institutional Review Board at the University of Alabama at Birmingham (Protocol #300010408).

### Patient Selection

Patients were excluded if their appointments occurred in the last three months of the dataset, because three-month post-appointment outcomes could not be observed. Patients were also excluded if they had a same-day ED visit or hospitalization on the date of a missed AICC appointment, as these visits likely represented direct clinical escalation rather than a failure to attend. The final analytic sample selection is detailed in Figure 1.

### Study Design and Methodologies

This retrospective cohort study employed a multivariable patient-level regression analysis to evaluate the effectiveness of the AICC in reducing ED visits and hospitalizations within three months of a patient’s AICC appointment.

### Primary Analysis

We used multivariable logistic regression as the primary analytical approach to evaluate the association between AICC appointment status (arrived versus missed) and the primary outcome of a three-month ED visit. A parallel logistic regression model assessed the secondary outcome of three-month hospitalization. All models adjusted for demographic, socioeconomic, and clinical characteristics to address potential confounding.

### Secondary Analyses for Robustness

To account for potential overdispersion and zero-inflation in the dependent variable, Poisson, negative binomial (NB), zero-inflated Poisson (ZIP), and zero-inflated negative binomial (ZINB) regression models were employed. These additional analyses provided complementary perspectives and tested the consistency of associations observed in primary analyses.

## RESULTS

### Patient Selection and Sample Characteristics

Our original dataset included 3,137 AICC appointments over the first three years of operations (May 21, 2020, to June 12, 2023). After applying exclusion criteria described above, our final analytic sample consisted of 2,698 appointments (Figure). Missing data were observed for sex in one patient (< 0.1%); all other variables had complete data.

The mean age was 51.5 years (24.0), and the sample was evenly split by sex (49.7% female). Most patients were White (58.2%) or Black (38.0%). Of the 2,698 appointments, 2,389 (88.5%) arrived and 309 (11.5%) were missed (cancelled or no-show). Among patients who attended their AICC appointment, none had an ED visit or hospitalization on the same day, indicating that all patients were managed without requiring same-day escalation to acute care.

### ED Visits

Table 1 presents the bivariate descriptive analysis results for three-month ED visits. Patients who missed their AICC appointment had a higher ED visit rate compared with those who arrived (47/309 [15.2%] vs 250/2,389 [10.5%]; risk difference, 4.75%; 95% CI, 0.56–8.93%; χ^2^ = 5.82; *P* = .02). Significant bivariate differences were also observed for age, race, sex, marital status, and diagnoses of asthma, anxiety, and depression (Table 1).

### Hospitalizations

Table 2 presents the bivariate descriptive analysis results for three-month hospitalizations. Patients who missed their AICC appointments had a higher hospitalization rate compared to those who arrived (165/309 [53.4%] vs 1,131/2,389 [47.3%]; risk difference, 6.06%; 95% CI, 0.14–11.97%; χ^2^ = 3.78; *P* = .05). Patients with a hospitalization were younger, had a higher proportion with Medicaid, and had a higher prevalence of multiple comorbidities (Table 2).

### Multivariable Regression Results

Table 3 presents the multivariable logistic regression results for ED visits and hospitalizations within three months of the AICC appointment.

### Emergency Department Visits

Missing an AICC appointment was significantly associated with 54% greater odds of an ED visit (OR, 1.54; 95% CI, 1.07–2.21; *P* = .02). Other significant predictors included prior ED visits (OR, 1.90; *P* < .001; prior hospitalizations (OR, 1.18; *P* = .03); Black race (OR, 1.74; *P* < .001); anxiety (OR, 1.57; *P* < .01); depression (OR, 1.76; *P* < .001); and cirrhosis (OR, 0.62; *P* = .02).

### Hospitalizations

Missing an AICC appointment was associated with 28% greater odds of hospitalization, but this was not statistically significant at the 95% confidence level (OR, 1.28; 95% CI, 1.00–1.64; *P* = .05). Significant predictors included prior ED visits (OR, 1.17; *P* = .04); prior hospitalizations (OR, 1.31; *P* < .001); younger age (OR, 0.99; *P* < .001); Medicare insurance compared with commercial insurance (OR, 1.30; *P* < .01); dialysis (OR, 0.58; *P* < .01); and heart failure (OR, 1.36; *P* < .01).

### Secondary Analyses

Results from secondary robustness analyses (Poisson, negative binomial, ZIP, and ZINB models) were consistent with the primary logistic regression results (Supplement Tables S1 and S2).

## DISCUSSION

This study evaluated the impact of a nurse-led AICC on reducing ED visits and hospitalizations within three months of an AICC appointment. By leveraging a multivariable analytical approach, this research assessed whether attending an AICC appointment influences healthcare use patterns and explored the clinic’s role within the broader healthcare system.

The findings strongly supported the first hypothesis, which proposed that patients adhering to AICC referrals would have fewer ED visits within three months of an AICC appointment. Patients who attended their AICC appointment were significantly less likely to visit the ED within three months compared to those who missed their appointment. This result highlights the critical importance of timely access to intermediate care in addressing acute healthcare needs and preventing escalation of conditions that often lead to emergency visits. These findings align with prior research emphasizing the role of enhanced access in reducing ED congestion and improving care outcomes.

In contrast, the second hypothesis, which suggested that AICC adherence would be associated with reduced hospitalizations, was not supported by the data. Although the point estimate directionally indicated a protective effect, this association was not statistically significant, and the robustness checks yielded similar findings. This lack of association may reflect the complexity of hospitalization decisions, which are influenced by factors beyond outpatient care access, particularly disease severity and comorbidities. Additionally, the weaker effect for hospitalization compared to ED visits might suggest that intermediate care has a more direct impact on avoiding acute exacerbations requiring ED-level intervention than on preventing inpatient admissions. Notably, no patients who attended their AICC appointment had an ED visit or hospitalization on the same day, suggesting that the AICC successfully managed all presenting patients without requiring same-day escalation to acute care. While our data do not capture whether patients were explicitly advised to return to the ED at a later date, the absence of same-day transfers supports the effectiveness of the AICC’s intermediate care model.

These results contribute to the theoretical understanding of access to care and systems theory. By addressing critical dimensions of access to care, such as availability, affordability, and accommodation, the AICC enhances patients’ ability to engage with the healthcare system at an intermediate level. This study demonstrated that the AICC mitigates barriers to care by offering same-day appointments, co-locating services, and employing multidisciplinary teams, which collectively improve patients’ healthcare experiences. These structural and operational enhancements reduce logistical barriers, such as scheduling difficulties and accessibility challenges, enabling patients to engage with the healthcare system more effectively.^21^,^22^ From a systems perspective, the AICC demonstrates how intermediate care clinics can act as vital nodes within the broader healthcare network, linking primary care and EDs to optimize resource allocation and patient flow. By absorbing nonurgent but high-risk cases that would otherwise escalate to ED visits, the AICC alleviates pressure on acute care services and enhances the overall efficiency of the system. However, the observed lack of a significant effect on hospitalizations highlights the complex interplay between systemic factors and individual patient trajectories,^23^–^25^ suggesting that hospitalization decisions are more influenced by clinical severity and comorbidities than by outpatient care availability. These findings underscore the need for integrated strategies that bridge gaps in both access and coordination, ensuring that intermediate care models like the AICC can address the diverse needs of patients while reducing systemic inefficiencies.

Black patients demonstrated higher odds of ED use even after controlling for demographic, socioeconomic, and clinical factors. While the AICC was associated with reduced ED visits overall, this finding suggests that the AICC model may not equally mitigate ED utilization across all racial groups. The AICC’s design, including same-day referral pathways, co-location with outpatient clinics, and lower copays relative to ED visits, may not sufficiently address the specific access barriers experienced by Black patients that contribute to higher ED use. Future research should explore how intermediate care models like the AICC can be tailored to more effectively reduce ED use among populations disproportionately affected by access barriers.

Patients with anxiety or depression also exhibited significantly higher odds of ED use, suggesting that the AICC model may benefit from incorporating mental health screening or integrated behavioral health services to better address the needs of these patients.^26^,^27^ Conversely, hospitalizations were more strongly associated with clinical factors, such as heart failure, pointing to the differentiated pathways driving ED versus inpatient service use. These patterns suggest that while the AICC can effectively address acute care needs that lead to ED visits, reducing hospitalizations may require complementary strategies targeting chronic disease management and care coordination.

Because the AICC model operates within existing outpatient infrastructure, it requires no additional facility construction. Based on the program’s internal operational report, the initial equipment and setup costs were approximately $100,000. Annual operating costs during early implementation totaled approximately $600,000, primarily attributable to staffing. For the fully developed future-state model, annual staffing costs are estimated at approximately $750,000 per year. The AICC was not always financially viable on a standalone basis, particularly during its early implementation period when referral volumes were limited by both the natural ramp-up of a new clinical program and the impact of the COVID-19 pandemic on outpatient operations. However, the financial assessment extends beyond direct AICC revenue. The AICC provides system-level benefits, including decompressing the ED, reducing avoidable admissions for low-acuity patients, and creating capacity for higher acuity patients, which positively impacts revenue across the organization. Since the study period ended, the AICC has continued to grow, currently averaging approximately 15 patients per day, further demonstrating the viability of the model. A formal cost-effectiveness analysis, including estimated savings from averted ED visits and hospitalizations, is an important area for future study.

## LIMITATIONS

This study has limitations. As a single-site study, the generalizability of these findings to other healthcare systems may be limited, particularly in regions with different patient populations, resource availability, or healthcare structures. Nevertheless, the single-site design allowed for a controlled evaluation with a clear emphasis on intermediate care, providing insights that can guide the adaptation of intermediate care models to similar settings. Additionally, while the study focused on short-term outcomes (three-month ED visits and hospitalizations), this time frame may not fully capture the long-term effects of AICC adherence on healthcare use or patient outcomes. However, three-month outcomes are widely accepted metrics to indicate the quality of care and align with operational priorities.^28^,^29^

Lastly, this study primarily relied on administrative data, which, while comprehensive, may not capture nuanced patient experiences or reasons for nonadherence to AICC referrals. Future studies incorporating qualitative methods or patient-reported outcomes could complement these findings by providing a deeper understanding of barriers to AICC use and its broader impact on care coordination.

## CONCLUSION

This study underscores the value of nurse-led intermediate care clinics like an ambulatory intermediate clinic in reducing ED use. However, the findings also suggest that their impact on hospitalization rates may be limited, emphasizing the need for complementary strategies to address inpatient admissions. As healthcare systems strive to address systemic inefficiencies, expanding and refining intermediate care models like the AICC could provide scalable solutions to balance care quality and efficiency. Future research should explore the long-term outcomes of nurse-led intermediate care clinics and their integration within broader healthcare networks, focusing on their potential to reduce disparities and enhance patient-centered care delivery.

## Supplementary Information



## Figures and Tables

**Figure:**
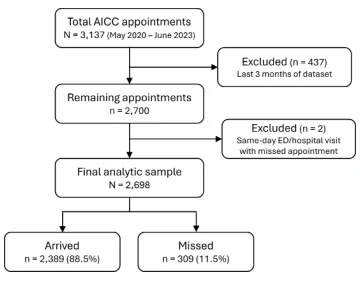
Patient selection flow diagram for this study. *AICC*, ambulatory intermediate care clinic; *ED*, emergency department.

**Table 1:** Univariate descriptive characteristics and bivariate comparisons for three-month emergency department visits among patients referred to the ambulatory intermediate care clinic in a study evaluating the impact of appointment attendance.

	All	No three-month ED visit	Yes three-month ED visit	*P* value
AICC Factor				
AICC appointment status				*P* = .02
Arrived	2,389 (88.5)	2,139 (89.1)	250 (84.2)	
Missed (cancelled / no show)	309 (11.5)	262 (10.9)	47 (15.8)	
Demographics and Social Determinants of Health				
Age, M (SD)	51.5 (24.0)	51.8 (23.9)	48.8 (24.4)	*P* < .001
Race/ethnicity, *n* (% of total)				*P* < .001
White	1,570 (58.2)	1,434 (59.7)	136 (45.8)	
Black	1,025 (38.0)	873 (36.4)	152 (51.2)	
Asian	40 (1.5)	34 (1.4)	6 (2.0)	
Hispanic or Latino	29 (1.1)	26 (1.1)	3 (1.0)	
Other	34 (1.3)	34 (1.4)	0 (0)	
Sex, *n* (% of total)				*P* < .001
Female	1,340 (49.7)	1,156 (48.2)	184 (62.0)	
Male	1,357 (50.3)	1,244 (51.8)	113 (38.0)	
Marital status, *n* (% of total)				*P* = .02
Married/life partner	1,345 (49.9)	1,223 (51.0)	122 (41.1)	
Single	774 (28.7)	668 (27.8)	106 (35.7)	
Divorced/separated	291 (10.8)	255 (10.6)	36 (12.1)	
Widowed	222 (8.2)	195 (8.1)	27 (9.1)	
Unknown	66 (2.4)	60 (2.5)	6 (2.0)	
Preferred language, *n* (% of total)				*P* = .92
English	2,609 (96.7)	2,320 (96.6)	289 (97.3)	
Spanish	14 (0.5)	12 (0.5)	2 (0.7)	
Other/unknown	75 (2.8)	69 (2.9)	6 (2.0)	
Payor type, *n* (% of total)				*P* = .50
Commercial	1,111 (41.2)	989 (41.2)	122 (41.1)	
Medicare	703 (26.1)	632 (26.3)	71 (23.9)	
Medicaid	773 (28.6)	685 (28.5)	88 (29.6)	
Uninsured	93 (3.4)	81 (3.4)	12 (4.0)	
Unknown	18 (0.7)	14 (0.6)	4 (1.4)	
Comorbidities				
Diabetes	1,151 (42.7)	1,030 (42.9)	121 (40.7)	*P* = .52
Myocardial infarction	411 (15.2)	358 (14.9)	53 (17.8)	*P* = .21
Coronary artery disease	891 (33.0)	803 (33.4)	88 (29.6)	*P* = .21
Chronic kidney disease	1,324 (49.1)	1,191 (49.6)	133 (44.8)	*P* = .13
Hypertension	1,658 (61.5)	1,475 (61.4)	183 (61.6)	*P* = 1.00
COPD	397 (14.7)	345 (14.4)	52 (17.5)	*P* = .18
Venous thromboembolism pulmonary embolism	549 (20.3)	488 (20.3)	61 (20.5)	*P =* .99
Asthma	297 (11.0)	252 (10.5)	45 (15.2)	*P* = .02
Anxiety	887 (32.9)	738 (30.7)	149 (50.2)	*P <* .001
Autoimmune disorder	319 (11.8)	278 (11.6)	41 (13.8)	*P* = .31
Depression	768 (28.5)	636 (26.5)	132 (44.4)	*P* < .001
Dyslipidemia	1,365 (50.6)	1,219 (50.8)	146 (49.2)	*P* = .64
Heart failure	875 (32.4)	765 (31.9)	110 (37.0)	*P* = .08

*AICC*, ambulatory intermediate care clinic; COPD, chronic obstructive pulmonary disease; *ED*, emergency department; *M*, mean; *SD*, standard deviation.

**Table 2:** Bivariate comparisons for three-month hospitalizations among patients referred to the ambulatory intermediate care clinic in a study evaluating the impact of clinic appointment attendance.

	No three-month hospitalization	Yes three-month hospitalization	*P* value
AICC Factor			
AICC appointment status			*P* = .05
Arrived	1,258 (52.7)	144 (46.6)	
Missed (cancelled / no show)	1,131 (47.3)	165 (53.4)	
Demographics and Social Determinants of Health			
Age, M (SD)	53.8 (21.0)	49.0 (26.7)	*P* < .001
Race/ethnicity, *n* (% of total)			*P* = .05
White	793 (56.6)	777 (60.0)	
Black	541 (38.6)	484 (37.3)	
Asian	25 (1.8)	15 (1.2)	
Hispanic or Latino	21 (1.5)	8 (0.6)	
Other	22 (1.5)	12 (0.9)	
Sex, *n* (% of total)		*P* = .12	
Female	718 (51.2)	623 (48.1)	
Male	684 (48.8)	673 (51.9)	
Marital status, *n* (% of total)			*P* = .12
Married/life partner	690 (49.2)	655 (50.6)	
Single	425 (30.3)	349 (26.9)	
Divorced/separated	151 (10.8)	140 (10.8)	
Widowed	100 (7.1)	122 (9.4)	
Unknown	36 (2.6)	30 (2.3)	
Preferred language, *n* (% of total)			*P* = .19
English	1,353 (96.5)	1,256 (96.9)	
Spanish	11 (0.8)	3 (0.2)	
Other/unknown	38 (2.7)	37 (2.9)	
Payor type, *n* (% of total)			*P* =.01
Commercial	598 (42.6)	513 (39.6)	
Medicare	366 (26.1)	337 (26.0)	
Medicaid	374 (26.7)	399 (30.8)	
Uninsured	49 (3.5)	44 (3.4)	
Unknown	15 (1.1)	3 (0.2)	
Comorbidities			
Diabetes	561 (40.0)	590 (45.5)	*P* < .01
Myocardial infarction	179 (12.8)	232 (17.9)	*P* < .001
Coronary artery disease	414 (29.5)	477 (36.8)	*P* < .001
Chronic kidney disease	668 (47.6)	656 (50.6)	*P* = .13
Hypertension	828 (59.1)	830 (64.0)	*P* < .01
COPD	176 (12.6)	221 (17.1)	*P* < .01
Venous thromboembolism pulmonary embolism	224 (16.0)	325 (25.1)	*P <* .001
Asthma	154 (11.0)	143 (11.0)	*P* = 1.00
Anxiety	433 (30.9)	454 (35.0)	*P =* .02
Autoimmune disorder	162 (11.6)	157 (12.1)	*P* = .70
Depression	382 (27.2)	386 (29.8)	*P* = .16
Dyslipidemia	661 (47.1)	704 (54.3)	*P* < .001
Heart failure	376 (26.8)	499 (38.5)	*P* < .001

*AICC*, ambulatory intermediate care clinic; COPD, chronic obstructive pulmonary disease; *M*, mean; *SD*, standard deviation.

**Table 3:** Multivariable logistic regression results for three-month emergency department visits and hospitalizations in a study assessing the association between ambulatory intermediate care clinic appointment attendance and subsequent healthcare use.

Variable	Three-month ED visit OR (95% CI)	Three-month hospitalization OR (95% CI)
Past three-month ED visit frequency	1.90*** (1.60, 2.26)	1.17* (1.01, 1.35)
Past three-month hospitalization frequency	1.18* (1.02, 1.36)	1.31*** (1.18, 1.44)
AICC appointment status		
Arrived	Reference	Reference
Missed (canceled or no show)	1.54* (1.07, 2.21)	1.28^†^ (1.00, 1.64)
Age in years	1.00 (0.99, 1.00)	0.99*** (0.99, 0.99)
Race		
White	Reference	Reference
Black	1.74*** (1.30, 2.34)	0.97 (0.80, 1.19)
Asian	1.70 (0.67, 4.34)	0.62 (0.31, 1.24)
Hispanic or Latino	1.46 (0.41, 5.21)	0.56 (0.24, 1.30)
Marital status		
Married/life partner	Reference	Reference
Divorced/separated	1.11 (0.73, 1.69)	0.97 (0.80, 1.19)
Single	1.02 (0.74, 1.41)	0.62 (0.31, 1.24)
Widowed	1.07 (0.64, 1.73)	0.56 (0.24, 1.30)
Primary insurer		
Commercial	Reference	Reference
Medicaid	1.03 (0.75, 1.41)	1.30** (1.07, 1.58)
Medicare	1.09 (0.78, 1.53)	1.17 (0.95, 1.43)
Uninsured	1.13 (0.56, 2.27)	1.02 (0.65, 1.61)
Medical history		
Diabetes mellitus	0.96 (0.73, 1.28)	1.15 (0.97, 1.37)
Chronic kidney disease	0.82 (0.57, 1.16)	1.10 (0.90, 1.34)
Hypertension	0.99 (0.71, 1.38)	0.99 (0.81, 1.21)
Anxiety	1.57** (1.16, 2.12)	0.99 (0.81, 1.21)
Cirrhosis	0.62* (0.41, 0.93)	1.11 (0.89, 1.39)
Depression	1.76*** (1.29, 2.40)	0.94 (0.77, 1.16)
Any dialysis	0.61^†^ (0.35, 1.06)	0.58** (0.41, 0.81)
Heart failure	1.39^†^ (0.97, 2.00)	1.36** (1.09, 1.71)

All results reported to 2 decimal places.

*AICC*, ambulatory intermediate care clinic; *OR*, odds ratio.
