# Supplementary material for: Effectiveness of a Nurse-Led Ambulatory Care Clinic in Reducing Emergency Department Visits and Hospitalizations
Source: West J Emerg Med. 2026 Sep 5;27(5):1199–207. doi: 10.5811/westjem.53190 (PMC13660612; doi:10.5811/westjem.53190)
Supplement: Supplementary file 1 [file wjem-27-1199-s001.docx]

***Supplementary Material***

*Effectiveness of a Nurse-Led Ambulatory Care Transition Clinic in Reducing Emergency Department Visits and Hospitalizations*

Table S1. Secondary robustness analyses (Poisson, negative binomial, and zero-inflated models) for 3-month ED visits in a study evaluating the impact of AICC appointment attendance.

| Variable | Poisson | NB | ZIP | ZINB |
| --- | --- | --- | --- | --- |
|  | RR (95% CI) | RR (95% CI) | RR (95% CI) | RR (95% CI) |
| Past 3-month ED visit frequency | **1.74*****  **(1.60, 1.90)** | **1.93*****  **(1.68, 2.21)** | **1.24*****  **(1.10, 1.40)** | **1.24*****  **(1.10, 1.40)** |
| Past 3-month hospitalization frequency | 1.11†  (0.996, 1.226) | 1.14†  (1.00, 1.30) | 1.01  (0.88, 1.17) | 1.01  (0.88, 1.17) |
| AICC appointment status | | | | |
| Arrived | Reference | | | |
| Missed (canceled or no show) | **1.57*****  **(1.21, 2.03)** | **1.45***  **(1.04, 2.04)** | **1.76****  **(1.15, 2.68)** | **1.76****  **(1.15, 2.68)** |
| Age in years | 1.00  (0.99, 1.000) | 1.00  (0.99, 1.00) | 1.00†  (0.99, 1.00) | 1.00†  (0.99, 1.00) |
| Race | | | | |
| White | Reference | | | |
| Black | **1.51*****  **(1.20, 1.88)** | **1.59****  **(1.21, 2.10)** | **1.41***  **(1.08, 1.85)** | **1.40***  **(1.07, 1.83)** |
| Asian | 1.35  (0.63, 2.91) | 1.69  (0.71, 4.04) | 1.33  (0.54, 3.27) | 1.33  (0.54, 3.27) |
| Hispanic or Latino | 0.95  (0.30, 3.05) | 0.90  (0.23, 3.57) | 0.70  (0.21, 2.37) | 1.42  (0.42, 4.88) |
| Marital status | | | | |
| Married/life partner | Reference | | | |
| Divorced/separated | 1.25  (0.90, 1.73) | 1.16  (0.78, 1.73) | 1.23  (0.84, 1.80) | 1.23  (0.84, 1.80) |
| Single | 1.22†  (0.96, 1.57) | 1.08  (0.80, 1.46) | 1.17  (0.87, 1.57) | 1.17  (0.87, 1.57) |
| Widowed | **1.59****  **(1.14, 2.22)** | 1.49†  (0.98, 2.27) | **1.68***  **(1.11, 2.57)** | **1.68***  **(1.11, 2.57)** |
| Primary insurer | | | | |
| Commercial | Reference | | | |
| Medicaid | 1.14  (0.90, 1.44) | 1.12  (0.84, 1.50) | 1.16  (0.89, 1.52) | 1.16  (0.89, 1.52) |
| Medicare | 1.14  (0.87, 1.49) | 1.15  (0.84, 1.59) | 1.13  (0.82, 1.56) | 1.13  (0.82, 1.56) |
| Uninsured | 1.24  (0.76, 2.01) | 1.26  (0.67, 2.38) | 1.33  (0.75, 2.36) | 1.33  (0.75, 2.36) |
| Medical history | | | | |
| Diabetes mellitus | 0.98  (0.79, 1.22) | 1.06  (0.81, 1.38) | 1.14  (0.87, 1.49) | 1.14  (0.87, 1.49) |
| Chronic kidney disease | 0.82  (0.57, 1.16) | 0.79  (0.57, 1.09) | **0.67***  **(0.48, 0.93)** | **0.67***  **(0.48, 0.93)** |
| Hypertension | 1.04  (0.81, 1.35) | 1.01  (0.75, 1.38) | 1.07  (0.79, 1.46) | 1.07  (0.79, 1.46) |
| Anxiety | **1.47****  **(1.16, 1.86)** | **1.40***  **(1.05, 1.87)** | 1.34†  (1.00, 1.79) | 1.34†  (1.00, 1.79) |
| Cirrhosis | **1.47****  **(1.16, 1.86)** | 0.78  (0.55, 1.13) | 0.82  (0.57, 1.20) | 0.82  (0.57, 1.20) |
| Depression | **1.51*****  **(1.18, 1.92)** | **1.66*****  **(1.24, 2.23)** | **1.52****  **(1.129, 2.048)** | **1.52****  **(1.13, 2.05)** |
| Any dialysis | **0.64***  **(0.41, 0.98)** | 0.68  (0.40, 1.14) | 0.68  (0.41, 1.13) | 0.68  (0.41, 1.13) |
| Heart failure | 1.17  (0.89, 1.55) | 1.12  (0.79, 1.58) | 1.18  (0.85, 1.64) | 1.18  (0.85, 1.64) |
| Zero-inflation part | | | | |
|  |  |  | OR (95% CI) | OR (95% CI) |
| Past 3-month ED visit frequency |  |  | **0.38*****  **(0.24, 0.59)** | **0.38*****  **(0.24, 0.59)** |
| Past 3-month hospitalization frequency |  |  | 0.86  (0.69, 1.07) | 0.86  (0.69, 1.07) |
| AICC appointment status | | | | |
| Arrived |  |  | Reference | |
| Missed (canceled or no-show) |  |  | 1.13  (0.62, 2.04) | 1.13  (0.62, 2.04) |
| NB, negative binomial; ZIP, zero-inflated Poisson; ZINB, zero-inflated negative binomial; AICC, ambulatory intermediate care clinic; ED, emergency department; RR, rate ratio; OR, odds ratio; CI, confidence interval. All results reported to 2 decimal places. | | | | |

Table S2. Secondary robustness analyses (Poisson, negative binomial, and zero-inflated models) for 3-month hospitalizations in a study evaluating the impact of AICC appointment attendance.

| Variable | Poisson | NB | ZIP | ZINB |
| --- | --- | --- | --- | --- |
|  | RR (95% CI) | RR (95% CI) | RR (95% CI) | RR (95% CI) |
| Past 3-month ED visit frequency | **1.08***  **(1.00, 1.16)** | 1.08†  (1.00, 1.16) | 1.07†  (0.99, 1.15) | 1.07†  (1.00, 1.16) |
| Past 3-month hospitalization frequency | **1.21*****  **(1.15, 1.26)** | **1.21*****  **(1.15, 1.27)** | **1.23*****  **(1.16, 1.29)** | **1.22*****  **(1.16, 1.29)** |
| AICC appointment status | | | | |
| Arrived | Reference | | | |
| Missed (canceled or no-show) | 1.12  (0.98, 1.29) | 1.12  (0.97, 1.29) | 1.10  (0.95, 1.27) | 1.11  (0.96, 1.28) |
| Age in years | **0.99*****  **(0.99, 0.99)** | **0.99*****  **(0.99, 1.00)** | **1.00*****  **(0.99, 1.00)** | **0.99*****  **(0.99, 1.00)** |
| Race | | | | |
| White |  |  |  |  |
| Black | 0.99  (0.89, 1.11) | 0.99  (0.89, 1.11) | 0.99  (0.89, 1.11) | 0.99  (0.89, 1.11) |
| Asian | 0.64†  (0.41, 0.99) | 0.64†  (0.41, 0.99) | 0.64†  (0.41, 0.99) | 0.64†  (0.408, 0.99) |
| Hispanic or Latino | 0.61  (0.33, 1.13) | 0.61  (0.33, 1.14) | 0.62  (0.33, 1.15) | 0.61  (0.33, 1.14) |
| Marital status | | | | |
| Married/life partner | Reference | | | |
| Divorced/separated | 0.98  (0.84, 1.15) | 0.99  (0.84, 1.16) | 0.98  (0.84, 1.15) | 0.98  (0.84, 1.15) |
| Single | **0.86***  **(0.76, 0.97)** | **0.86***  **(0.76, 0.98)** | **0.86***  **(0.76, 0.97)** | **0.86***  **(0.76, 0.97)** |
| Widowed | **1.19***  **(1.01, 1.40)** | **1.19***  **(1.00, 1.40)** | **1.18***  **(1.00, 1.39)** | 1.18†  (1.00, 1.39) |
| Primary insurer | | | | |
| Commercial | Reference | | | |
| Medicaid | 1.09  (0.98, 1.22) | 1.09  (0.97, 1.23) | 1.10  (0.98, 1.23) | 1.10  (0.98, 1.23) |
| Medicare | 1.01  (0.89, 1.14) | 1.01  (0.89, 1.14) | 1.01  (0.89, 1.14) | 1.01  (0.89, 1.14) |
| Uninsured | 1.15  (0.90, 1.46) | 1.15  (0.90, 1.48) | 1.15  (0.90, 1.47) | 1.15  (0.90, 1.48) |
| Medical history | | | | |
| Diabetes mellitus | 1.10†  (1.00, 1.22) | 1.10†  (0.99, 1.22) | 1.10†  (0.99, 1.21) | 1.10†  (0.99, 1.21) |
| Chronic kidney disease | 1.06  (0.94, 1.20) | 1.06  (0.94, 1.20) | 1.06  (0.94, 1.20) | 1.06  (0.94, 1.20) |
| Hypertension | 0.97  (0.86, 1.09) | 0.97  (0.86, 1.09) | 0.97  (0.86, 1.09) | 0.97  (0.86, 1.10) |
| Anxiety | 0.99  (0.88, 1.11) | 0.99  (0.88, 1.11) | 0.98  (0.88, 1.10) | 0.98  (0.88, 1.10) |
| Cirrhosis | 1.09  (0.97, 1.24) | 1.10  (0.97, 1.24) | 1.09  (0.96, 1.23) | 1.09  (0.96, 1.24) |
| Depression | 1.05  (0.94, 1.18) | 1.05  (0.93, 1.18) | 1.05  (0.94, 1.18) | 1.05  (0.93, 1.18) |
| Any dialysis | **0.64*****  **(0.53, 0.77)** | **0.64*****  **(0.52, 0.77)** | **0.63*****  **(0.52, 0.76)** | **0.63*****  **(0.52, 0.77)** |
| Heart failure | **1.23****  **(1.08, 1.39)** | **1.23****  **(1.08, 1.40)** | **1.23****  **(1.08, 1.39)** | **1.23****  **(1.08, 1.40)** |
| Zero-inflation part | | | | |
|  |  |  | OR (95% CI) | OR (95% CI) |
| Past 3-month ED visit frequency |  |  | 0.53  (0.03, 8.83) | 1.20  (0.50, 2.80) |
| Past 3-month hospitalization frequency |  |  | 1.79  (0.74, 4.30) | 2.00  (0.90, 4.44) |
| AICC appointment status | | | | |
| Arrived |  |  | Reference | |
| Missed (canceled or no-show) |  |  | 0.00  (0.00, 59.77) | 0.50  (0.10, 2.50) |
| NB, negative binomial; ZIP, zero-inflated Poisson; ZINB, zero-inflated negative binomial; AICC, ambulatory intermediate care clinic; ED, emergency department; RR, rate ratio; OR, odds ratio; CI, confidence interval. All results reported to 2 decimal places. | | | | |
